# Ligation of Na, K ATPase β3 subunit on monocytes by a specific monoclonal antibody mediates T cell hypofunction

**DOI:** 10.1371/journal.pone.0199717

**Published:** 2018-06-25

**Authors:** Nuchjira Takheaw, Witida Laopajon, Sirirat Surinkaew, Saichit Khummuang, Supansa Pata, Watchara Kasinrerk

**Affiliations:** 1 Division of Clinical Immunology, Department of Medical Technology, Faculty of Associated Medical Sciences, Chiang Mai University, Chiang Mai, Thailand; 2 Biomedical Technology Research Center, National Center for Genetic Engineering and Biotechnology, National Science and Technology Development Agency at the Faculty of Associated Medical Sciences, Chiang Mai University, Chiang Mai, Thailand; Emory University School of Medicine, UNITED STATES

## Abstract

T cells play a crucial role in orchestrating body immune responses. T cell hyperfunction, however, leads to inflammation and induction of autoimmune diseases. Understanding of T cell regulation mechanisms and successful modulation of T cell responses is beneficial in treatment of disease associated to T cell hyperresponsiveness. Our previous study indicated that monoclonal antibody (mAb) P-3E10, a mAb to Na, K ATPase β3 subunit, inhibited anti-CD3-induced PBMC proliferation. In the current study, we further investigated the mechanism of mAb P-3E10 in the induction of T cell hypofunction. We demonstrated that mAb P-3E10 decreased T cell proliferation and Th1, Th2 and Th17 cytokine production. Monocytes were the cells playing a key role in mediation of mAb P-3E10 induced T cell hypofunction. The inhibition of T cell activation by mAb P-3E10 required cell contact between monocytes and T cells. The mAb P-3E10 induced the down-expression level of MHC class II and CD86 and increased IL-6, IL-10 and TNF-α production of monocytes. We concluded that ligation of the Na, K ATPase β3 subunit on monocytes by mAb P-3E10 arbitrated T cell hypofunction. This mAb might be a promising novel immunotherapeutic antibody for the treatment of hyperresponsive T cell associated diseases.

## Introduction

T cells are the cells that function as a key regulator in the immune responses to defeat pathogens, but maintain self-tolerance [1]. The activation of naïve T cells requires at least two signals. The first signal is delivered by TCR-CD3 complexes upon the interaction between TCR and peptide-MHC molecule presented by antigen presenting cells (APCs). The second signal is generated by the co-stimulatory molecules. Only the first signal received without the second signal results in unresponsiveness or anergy state of T cells [2, 3]. Activation of CD4^+^ T cells is programmed by specific polarizing cytokines released from APCs and leading to the differentiation of T cells into a variety of specialized effector cells. These effector T cells are effectively combatting a variety of pathogens by secreting distinct cytokines [4]. According to the crucial role of T cells in immunoregulation, prolonged T cell activation may lead to severe inflammation and be involved in the occurrence of autoimmune diseases [5–8]. Reduction of hyperresponsiveness of T cells is important for the body in order to control its adverse effects. Several mechanisms for the persuasion of T cell hypofunction have been introduced and proposed for clinical intervention. For instance, the short-term blockade of the interaction of co-stimulatory molecules between T cells and APCs was demonstrated to downregulate T cell function and suggested as a promising immunotherapeutic strategy for autoimmune diseases [9–12]. Identification and characterization of novel molecular mechanisms underlying negative immune regulation might provide new targets for immunotherapy.

In our laboratory, a monoclonal antibody (mAb) named P-3E10 was generated. The molecule recognized by mAb P-3E10 was identified as Na, K ATPase β3 subunit or CD298 [13, 14]. Inhibition of T cell proliferation, CD25 expression and cell cycle arrest by mAb P-3E10 have been demonstrated [13, 15]. In this study, we further investigated the mechanisms involving the inhibitory effect of mAb P-3E10 on T cell activation. Our results demonstrated that the ligation of Na, K ATPase β3 subunit on monocytes by mAb P-3E10 is partaken in the inhibition of T cell activation. Our finding suggested a novel mechanism which controls T cell functions through Na, K ATPase β3 subunit on monocytes. We speculated that mAb P-3E10 might be a promising mAb to induce hypofunction of T cells and eventually might be applicable to immune-mediated therapy for severe inflammation and autoimmune diseases.

## Materials and methods

### Antibodies and reagents

Anti-Na, K ATPase β3 subunit mAb clone P-3E10 (IgG2a isotype) and anti-bacteriophage protein mAb clone 13M (IgG2a) were produced in our laboratory [13, 16]. Anti-CD3ε mAb (clone OKT3) was purchased from Ortho Pharmaceuticals (Raritan, NJ, USA). Anti-CD28 mAb (clone L293), FITC-labeled anti-CD3 mAb and PE-conjugated anti-CD25 mAb were obtained from BD Bioscience (San Jose, CA, USA). PerCP-conjugated anti-CD3 mAb, PerCP-labeled anti-CD14 mAb and PE-conjugated anti-human cytokine antibodies (anti-IL-2, IFN-γ, IL-4, IL-10, IL-17, TNF-α, IL-6) were purchased from BioLegend (San Diego, CA, USA). FITC-labeled anti-CD86, HLA-DR and HLA-ABC mAbs, PE-conjugated anti-CD69 mAb and mouse IgG1-PE isotype control antibody were purchased from ImmunoTools (Friesoythe, Germany). Percoll reagent was purchased from Amersham Biosciences (Uppsala, Sweden). Brefeldin A and Monensin were purchased from Sigma-Aldrich (St. Louis, MO, USA). Saponin was obtained from Amresco (Solon, OH, USA).

### Cells

THP-1 cells (a human monocytic cell line) [13] were maintained in RPMI-1640 medium supplemented with 10% fetal bovine serum (FBS) (Gibco, Grand Island, NY), 40 μg/mL Gentamycin and 2.5 μg/mL Amphotericin B and incubated in humidified atmosphere containing 5% CO_2_ at 37°C.

Peripheral blood mononuclear cells (PBMCs) were isolated from heparinized whole blood of healthy individuals using Ficoll-Hypaque gradient centrifugation (IsoPrep, Robbins Scientific Corporation, Sunnyvale, CA, USA). For preparation of monocyte-depleted PBMCs, the monocytes were depleted out from PBMCs by Percoll density gradient centrifugation. In brief, PBMCs were suspended in PBS and then layered above Percoll solution (48.5% Percoll and 0.16 M NaCl in ddH_2_O). After centrifugation at 865×*g* at room temperature for 40 min, the enriched monocyte layer and Percoll solution were discarded. Monocyte-depleted PBMCs were collected and washed with PBS three times. CD14^+^ monocyte contamination was determined as <2% by flow cytometry using the FACSort flow cytometer (BD Biosciences). CD3^+^ T cells and CD14^+^ monocytes were purified from human PBMCs by magnetic antibody cell sorting (MACS) system using Pan T Cell Isolation Kit and Pan Monocyte Isolation Kit (Miltenyi Biotec, Bergisch-Gladbach, Germany), respectively. The purity of CD3^+^ T cells and CD14^+^ monocytes was confirmed by flow cytometry and >95% purity was obtained. The study was approved by the ethics committees of the Faculty of Associated Medical Sciences, Chiang Mai University (AMSEC-60EX-022). Written informed consent was obtained from all studied subjects.

### Production and characterization of monoclonal antibody P-3E10

The mAb P-3E10 producing hybridoma cells were cultured in Iscove's Modified Dulbecco's Media (IMDM) supplement with 10% fetal bovine serum (FBS), 40 μg/mL gentamycin and 2.5 μg/mL amphotericin B and then adapted to grow in hybridoma serum free media (H-SFM; Gibco) containing the same antibiotics at 37°C in a 5% CO_2_ incubator. After adaptation, the hybridoma cells (4x10^6^ cells) were cultured in 10 mL of H-SFM for 4 days and culture supernatants were collected. Culture supernatants containing mAb P-3E10 were purified using affinity chromatography on HiTrap Protein G HP (GE Healthcare, Uppsala, Sweden). To determine the specificity of the purified mAb P-3E10, wild type BW5147 mouse thymoma cells and Na, K ATPase β3 subunit expressing BW5147 cells [13] were used for immunofluorescence staining. Wild type and Na, K ATPase β3 subunit expressing BW5147 cells were stained with purified mAb P-3E10 and mAb 13M (isotype-matched control mAb) at a final concentration of 10 μg/mL followed by FITC-conjugated goat anti-mouse IgG antibody (Millipore, Billerica, MA, USA). The specificity of mAb P-3E10 was analyzed by flow cytometry (BD AccuriTM C6, BD Biosciences) and shown in Supporting Information (S1A Fig). PBMCs were also stained with the purified mAb P-3E10. To block nonspecific Fc receptor-mediated binding of mAb, cells were pre-incubated for 30 min at 4°C with 10% human AB serum before staining. Cells were then stained with purified mAb P-3E10 and mAb 13M (isotype-matched control mAb) at a final concentration of 10 μg/mL followed by Alexa Fluor 488-conjugated goat anti-mouse IgG antibody (Invitrogen, Carlsbad, CA, USA). The specificity of mAb P-3E10 was analyzed by flow cytometry and shown in Supporting Information (S1B Fig).

### Cell proliferation assay

T cell proliferation assay was performed by the carboxyfluorescein succinimidyl ester (CFSE) dilution technique. PBMCs, monocyte-depleted PBMCs, or purified CD3^+^ T cells were labeled with CFSE (Sigma-Aldrich, St. Louis). Briefly, cells were suspended in PBS at 1x10^7^ cells/mL and incubated with 0.5 μM final concentration of CFSE for 10 min at 37°C. Afterward, the excess CFSE was quenched with cold 10% FBS-supplemented RPMI. Cells were washed two times and re-suspended in 10% FBS-supplemented RPMI. The CFSE-labeled PBMCs were plated at a density of 1×10^6^ cells/mL in a 96-well plate with or without immobilized anti-CD3 mAb clone OKT3 (25 ng/mL). The CFSE-labeled monocyte-depleted PBMCs and CFSE-labeled purified CD3^+^ T cells were plated with or without immobilized anti-CD3 mAb clone OKT3 (50 ng/mL) plus soluble anti-CD28 mAb (100 ng/mL). The cells were then cultured in the presence or absence of purified mAb P-3E10 or isotype matched control mAb.

For co-culture condition, purified CD3^+^ T cells and CD14^+^ monocytes were co-plated at 2.5:1 ratio either in a 96-well flat-bottom plate (Corning®, NY, USA) coated with mAb OKT3 or in separate compartments of a 96-well plate harboring a 0.4 μm porous polycarbonate membrane Transwell (Corning®, NY, USA), containing monocytes in the upper chamber and T cells in the lower chamber coated with anti-CD3 mAb (OKT3) plus soluble anti-CD28 mAb.

For mAb pre-pulsed monocyte co-culture, purified CD14^+^ monocytes were pre-incubated with mAb P-3E10 or isotype-matched control mAb at a concentration of 20 μg/mL for 30 min at room temperature. Cells were washed three times to remove unbound mAbs. Antibody pre-pulsed monocytes were co-cultured with purified T cells at 1:2.5 ratio in an OKT3-immobilized plate. The cells were incubated at 37°C in 5% CO_2_ incubator. After 5 days of cultivation, cells were harvested to measure the reduction of CFSE by flow cytometry (FACSort or BD Accuri^TM^ C6, BD Biosciences). The data was analyzed by FlowJo software.

### T cell activation marker analysis

THP-1 cells at a density of 5×10^5^ cells/mL were pre-pulsed with mAb P-3E10 or isotype-matched control mAb at a concentration of 10 μg/mL for 30 min at room temperature. After unbound mAbs were removed, mAb pre-pulsed THP-1 cells were co-cultured with OKT3-activated PBMCs at 1:4 ratio for 18 h at 37°C in 5% CO_2_ incubator. Harvested cells were fixed with 4% paraformaldehyde then permeabilized with 0.1% saponin in PBS containing 10% FBS-0.02% NaN_3_. After that, cells were stained with PerCP-conjugated anti-CD3 mAb and PE-conjugated anti-CD69 mAb, PE-anti-CD25 mAb or mouse IgG isotype control mAb. The stained cells were determined by flow cytometry (FACSort, BD Biosciences) and analyzed by FlowJo software.

### Intracellular cytokine analysis

PBMCs were cultured in a 24-well plate at a density of 4×10^6^ cells/mL with or without OKT3 (25 ng/mL) and soluble anti-CD28 mAb (25 ng/mL) in the presence or absence of the mAb P-3E10 or isotype-matched control mAb (20 μg/mL). After cultivation for 1 h, protein transport inhibitors (1 μg/mL Brefeldin A and 1μM Monensin) were added into each culture condition and continuously incubated for 5 h at 37°C in 5%CO_2_ incubator. The harvested cells were fixed with 4% paraformaldehyde, permeabilized with 0.1% saponin in PBS containing 10% FBS-0.02% NaN_3_. FITC-labeled anti-CD3 and PerCP-labeled anti-CD14 antibodies were mixed with each PE-conjugated anti-human cytokine antibody (anti- IFN-γ, IL-2, IL-4, IL-6, IL-10, IL-17 or TNF-α) or PE-IgG1 isotype-matched control antibody. The intracellular cytokines were determined by flow cytometry (FACSort, BD Biosciences) and analyzed by FlowJo software.

### Surface MHC class I, MHC class II and CD86 analysis

PBMCs were cultured in a 24-well plate at a density of 1.25×10^6^ cells/mL in 25 ng/mL of immobilized OKT3 in combination with mAb P-3E10 (20 μg/mL), isotype-matched control mAb (20 μg/mL) or without mAb. The cells were cultured for 18 h at 37°C in a 5% CO_2_ incubator. Cells were collected and surface stained with PerCP-conjugated anti-CD14 mAb and FITC-labeled anti-MHC class I (HLA-ABC), MHC class II (HLA-DR), CD86 or IgG isotype-matched control mAb. The cells were measured using a FACSort or BD Accuri^TM^ C6 flow cytometer (BD Biosciences) and analyzed by FlowJo software.

### Statistical analysis

All data are expressed as mean ± standard error (SEM). GraphPad Prism version 7 was used to perform all statistical analyses. Unpaired *t*-test, One-way or Two-way analysis of variance (ANOVA) was used. A *P* < 0.05 was considered statistically significant.

## Results

### Ligation of Na, K ATPase β3 subunit by mAb P-3E10 inhibits T cell activation

**Fig 1:**
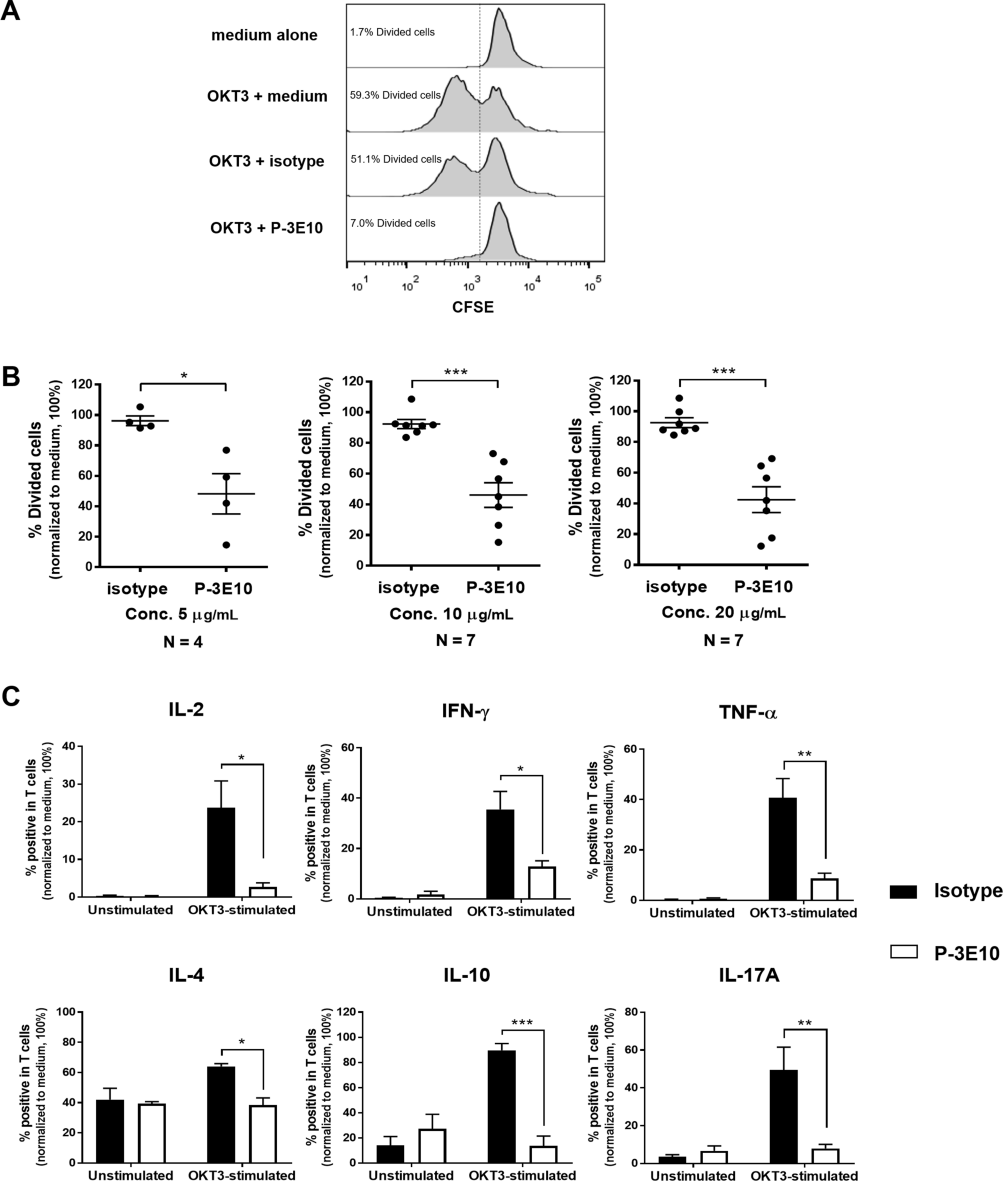
The inhibition of T cell activation by mAb P-3E10. (A) PBMCs were activated with anti-CD3 mAb OKT3 or kept unstimulated in the presence of mAb P-3E10 or mAb 13M (isotype-matched control mAb) or medium alone. Flow cytometric data were expressed in histograms showing the percentage of divided cells in each condition using CFSE proliferation assay. (B) The percentage of divided cells upon OKT3-induced PBMC proliferation in the presence of indicated concentrations of mAb P-3E10 or isotype-matched control mAb is shown. Each individual data was normalized relative to its medium control as 100%. Unpaired *t*-test was used for comparison, *P<0.05. ***P<0.001. (C) After stimulation as was described in (A), CD3^+^ T cells were gated. The percentage of the indicated cytokine producing T cells was determined and is shown as mean ± SE from three individuals. The data were exhibited as the percentage of cytokine positive cells by normalization to medium control of OKT3-stimulated condition as 100%. Two-way ANOVA followed by Tukey’s test was used for comparison, *P<0.05. **P<0.01. ***P<0.001. The flow cytometric data were illustrated in the Supporting Information (S2 Fig).

As shown in Fig 1A and 1B, activation of PBMCs with anti-CD3 mAb (OKT3) in the presence of mAb P-3E10 at concentrations of 5, 10 and 20 μg/mL significantly inhibited cell proliferation compared to isotype-matched control mAb.

Th1 cytokines (IL-2, IFN-γ and TNF-α), Th2 cytokines (IL-4 and IL-10) and Th17 cytokine (IL-17A) were intracellularly measured in activated T cells. Upon OKT3 activation, the number of T cells producing Th1, Th2 and Th17 cytokines was increased compared to un-stimulated condition. All cytokines produced by activated T cells were dramatically inhibited by mAb P-3E10 in comparison to isotype-matched control mAb (Fig 1C). Our results demonstrated that mAb P-3E10 has the ability to impair T cell responses, in both cell proliferation and cytokine production. The Th1, Th2 and Th17 cytokines were inhibited by mAb P-3E10.

### Monocytes play a major role in T cell hypofunction by mAb P-3E10

**Fig 2:**
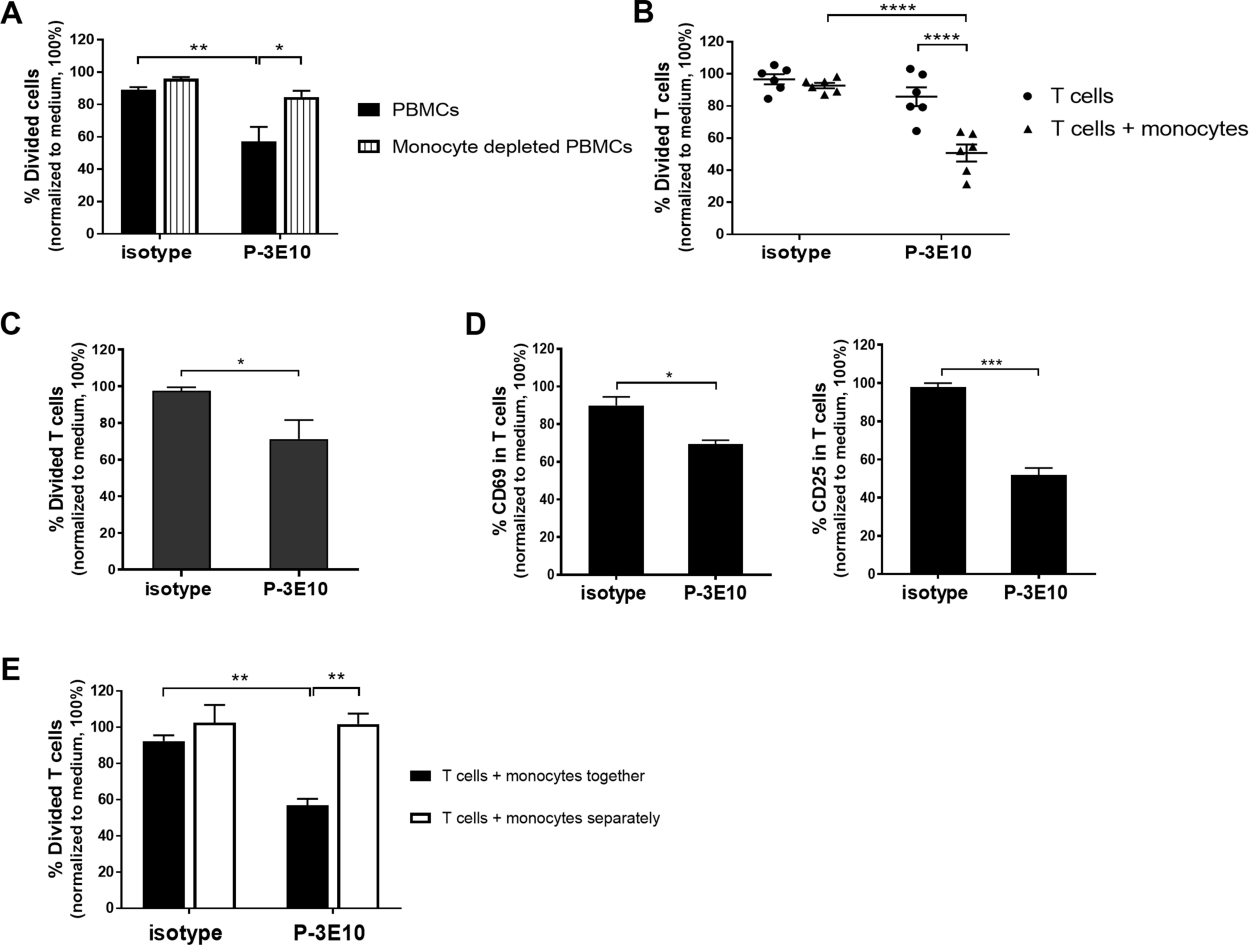
Ligation of monocytes by mAb P-3E10 regulates T cell activation. (A) PBMCs and monocyte-depleted PBMCs were activated with anti-CD3 mAb in the absence (medium control) or presence of mAb P-3E10 or isotype-matched control mAb. The percentage of divided cells at the indicated conditions is shown (*n* = 3). Two-way ANOVA followed by Tukey’s test was used for comparison. *P<0.05. **P<0.01. (B) Purified T cells and purified T cells co-cultured with autologous purified monocytes were activated with anti-CD3 mAb in the absence or presence of mAb P-3E10 or isotype-matched control mAb. The percentage of divided T cells at the indicated conditions is shown (*n* = 6). Two-way ANOVA followed by Tukey’s test was used for comparison. ****P<0.0001. (C) Monocytes were pre-pulsed with mAb P-3E10 or isotype-matched control mAb before adding to purified T cells. Cells were activated with anti-CD3 mAb and the percentage of divided T cells in the indicated conditions is shown (*n* = 6). (D) THP1-cells were pre-pulsed with mAb P-3E10 or isotype-matched control mAb. The pre-pulsed THP1 cells were co-cultured with PBMCs and activated with anti-CD3 mAb. CD3^+^ T cells were gated and % CD69 and % CD25 expressing T cells in the indicated conditions are shown (*n* = 3). All data was normalized to medium control as 100%. Unpaired *t*-test was used in the comparison. *P<0.05. ***P<0.001. (E) Purified T cells were co-cultured with autologous purified monocytes. Upon stimulation with anti-CD3 mAb in the presence or absence of mAb P-3E10 or isotype-matched control mAb, cells were cultured either in the same well or in separate compartments in a 96-transwell plate. The percentage of divided T cells at the indicated conditions is shown (*n* = 3). Two-way ANOVA followed by Tukey’s test was performed. **P<0.01. The flow cytometric data were illustrated in the Supporting Information (S3 Fig).

According to the aforementioned results, the inhibitory effect of mAb P-3E10 was observed in PBMC activation. PBMCs is composed of two major populations: lymphocytes and monocytes. We raised the question whether the effect of mAb P-3E10 on T cell activation occurs through the ligation of Na, K ATPase β3 subunit expressed on T cells or on monocytes. We then depleted monocytes out from PBMCs and further investigated the effect of mAb P-3E10 on OKT3 stimulated monocyte-depleted PBMCs compared to untouched PBMCs. The results showed that the inhibiting effect of mAb P-3E10 was diminished in monocyte-depleted PBMC (Fig 2A) suggesting that monocytes play a role in the induction of T cell hypofunction upon mAb P-3E10 ligation.

To confirm whether monocytes act as a key regulator in mAb P-3E10-impaired T cell activation, purified T cells and purified T cells co-cultured with autologous purified monocytes were activated in the presence of mAb P-3E10, isotype-matched control mAb or medium control. It was found that mAb P-3E10 inhibition effect could be observed in the T cell-monocyte co-culture, but not in the T cell alone (Fig 2B), indicating impairment of T cell activation by mAb P-3E10 is mediated through monocytes. We further investigated whether the ligation of Na, K ATPase β3 subunit on monocytes alone was sufficient for the inhibition of T cell activation. Purified monocytes were pre-incubated with mAb P-3E10 (or isotype-matched control mAb) to allow mAb binding to monocytes before added to purified T cells. The pre-pulsed monocytes with mAb P-3E10 significantly decreased T cell proliferation compared to isotype-matched control mAb pre-pulsed monocytes (Fig 2C). Furthermore, THP-1, a human monocytic cell line, was employed in the study. In correlation with the primary monocyte study, we found that mAb P-3E10 pre-pulsed THP-1 decreased the expression level of CD69 and CD25 activation markers on T cells’ surface compared to isotype control mAb pre-pulsed THP-1 (Fig 2D). These results specify that the ligation of Na, K ATPase β3 subunit by mAb P-3E10 on monocytes played a role in the inhibition of T cell functions.

To clarify whether the regulation of T cell activation by mAb P-3E10-activated monocytes involves a cell-cell interaction or is induced by secreted cytokines, we co-cultured T cells and autologous monocytes either together in a 96-well plate or in separate compartments of a 96-transwell plate. It was found that in contact between monocytes and T cells, mAb P-3E10 significantly decreased T cell proliferation (Fig 2E). In contrast, in the condition of co-cultured monocytes and T cells in separate compartments, mAb P-3E10 could not inhibit T cell proliferation (Fig 2E). These results suggested that cell-cell contact is required for the induction of T cell hypofunction; soluble factors secreted by mAb P-3E10-activated monocytes were not sufficient to limit T cell activation.

### Ligation of Na, K ATPase β3 subunit on monocytes by mAb P-3E10 downregulates MHC class II and CD86 expressions and upregulates IL-6, IL-10 and TNF-α production

**Fig 3:**
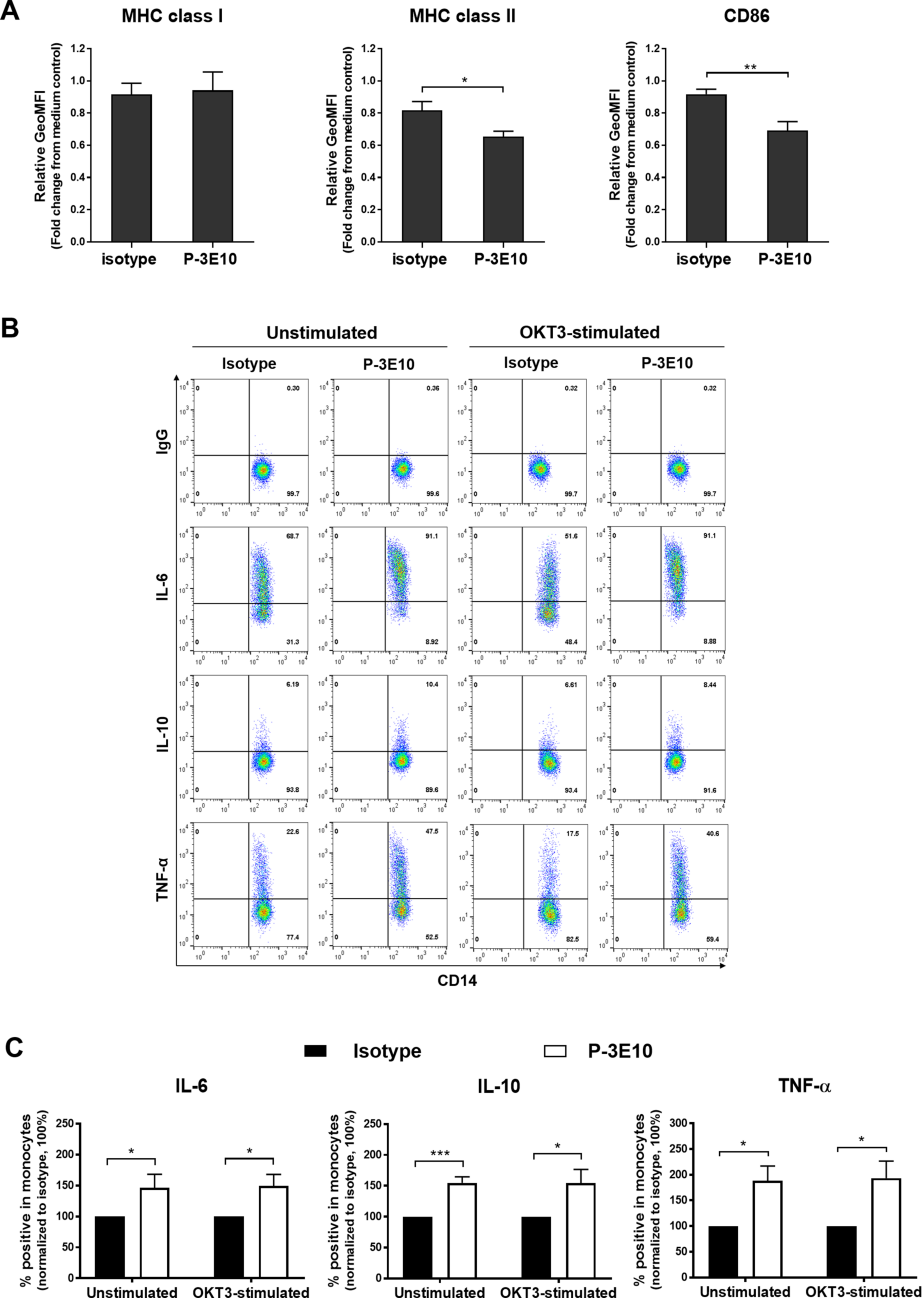
Ligation of Na, K ATPase β3 subunit on monocytes downregulates MHC class II and CD86 expressions and upregulates IL-6, IL-10 and TNF-α production. (A) PBMCs were stimulated with anti-CD3 mAb in the absence (medium control) or presence of mAb P-3E10 or isotype-matched control mAb. The surface expression levels of MHC class I (HLA-ABC) (*n* = 4), MHC class II (HLA-DR) (*n* = 3) and CD86 (*n* = 4) on CD14^+^ monocytes were determined by flow cytometry. The relative geometric mean fluorescence intensity (GeoMFI of specific marker mAb staining/GeoMFI of isotype-matched control mAb staining) was normalized to medium control as 1. Unpaired t-test was used in the comparison. *P<0.05. **P<0.01. (B) PBMCs were stimulated with anti-CD3 mAb or kept unstimulated in the absence (medium control) or presence of mAb P-3E10 or isotype-matched control mAb. The % IL-6, % IL-10 and % TNF-α producing CD14^+^ monocytes in the indicated conditions are shown as the representative result from one of the four individuals. (C) The data were analyzed as the percentage of cytokine positive cells (% specific cytokine staining—% isotype-matched control staining). The percentage of cytokine positive cells were normalized to isotype-matched mAb treated control of each activation condition as 100%. The data are expressed as mean ± SE (*n* = 4). Unpaired t-test was performed. *P<0.05. ***P<0.001. The flow cytometric data were illustrated in the Supporting Information (S4 Fig).

As mAb P-3E10-activated monocytes regulated T cells through cell-cell interactions, we further investigated the effect of mAb P-3E10 on MHC class I, MHC class II and CD86 expressions on monocyte surface. As shown in Fig 3A, MHC class II (HLA-DR) and CD86 expressions on monocytes were statistically significantly downregulated following treatment with mAb P-3E10. The expression of MHC class I (HLA-ABC), however, was not altered (Fig 3A). Several cytokines were known to be involved in modulating MHC and co-stimulatory molecules on monocytes. We compared the frequency of monocytes producing IL-6, IL-10 and TNF-α after incubation with mAb P-3E10 and isotype-matched control mAb. The percentages of IL-6, IL-10 and TNF-α producing monocytes were significantly increased by mAb P-3E10 in either in the presence or absence of anti-CD3 mAb (Fig 3B and 3C).

## Discussion

Na, K ATPase is a cell membrane-associated enzyme responsible for active transport of Na^+^ and K^+^ across the plasma membranes. The Na, K ATPase plays a vital role in transporting three sodium ions out of the cell in exchange for two potassium ions into the cell to maintain low concentrations of intracellular Na^+^ ions and high concentrations of intracellular K^+^ ions [17, 18]. It generates electrochemical ion gradients that are necessary for many cellular processes such as maintaining membrane potential, regulating cell volume and supporting secondary active transport [19–21]. The Na, K ATPase is composed of two subunits, α and β subunits. Currently, four α-subunits (α1, α2, α3, and α4) and three β-subunits (β1, β2, and β3) have been described in humans [22–25]. Within the Na, K ATPase complex, the α subunits are responsible for the catalytic activity and contain the binding sites for ATP, Na^+^ and K^+^. The β subunits, however, are structural proteins for stabilizing α subunits and function as chaperone to facilitate the correct folding of the α subunits [24, 26, 27]. A mAb term P-3E10 was generated in our laboratory. This mAb recognizes an epitope expressed on β3 subunit of Na, K ATPase [13] and was later defined as CD298 by the 8th HLDA Workshop [14].

Surprisingly, we demonstrated that the mAb P-3E10 could inhibit cell proliferation and Th1, Th2 and Th17 cytokine production upon stimulating PBMCs with anti-CD3 mAb OKT3. The inhibition effect of mAb P-3E10 was demonstrated to not be involved in the interference of Na, K ATPase activity [15]. As the inhibition effect of mAb P-3E10 was observed in OKT3 stimulated PBMCs, we raised the question whether monocytes or lymphocytes present in PBMCs play role in inducing T cell suppression under mAb P-3E10ligation. In order to address this question, we investigated the effect of mAb P-3E10 on monocyte-depleted PBMCs, purified T cells and T cells plus monocytes. Interestingly, mAb P-3E10 had no effect in attenuating T cell activation in the conditions lacking monocytes, both in monocyte-depleted PBMCs and purified T cells. However, mAb P-3E10 attenuated T cell activation in the presence of mAb P-3E10 pre-treated monocytes. The ligation of Na, K ATPase β3 subunit on monocytes, but not on T cells, is required for the negative regulation of T cell activation. Our finding demonstrated that monocytes are the cells playing a key role in the mediation of mAb P-3E10 induced T cell hypofunction. It is worthy to mention that the effect of mAb P-3E10 observed was not due to the potential binding of mAb Fc region to Fc receptor of monocytes. In this study, the isotype-matched control mAb was included and compared for determination of the effect of mAb P-3E10. The effects of mAb P-3E10 observed were demonstrated to be dependent on the mAb Fab binding to the recognized molecule.

Monocytes are the cells which play an important role in the regulation of T cell function [28, 29]. Several regulation mechanisms, involving either cell surface molecules or cytokines, have been documented for the monocyte function in the regulation of T cell activation [30–34]. We demonstrated that the ligation of Na, K ATPase β3 subunit on monocytes by mAb P-3E10 suppressed T cell activation involving cell-to-cell contact between T cells and monocytes; soluble factors alone were not sufficient to inhibit T cell function. Several molecules including MHC molecules and co-stimulatory molecules were demonstrated to be involved in the interaction between monocytes and T cells. We therefore investigated the expression of a set of surface molecules on monocytes, which are involved in T cell activation [35, 36], and whether their expression was altered during mAb P-3E10 treatment. The upregulation of monocyte co-stimulating molecules, MHC class II (HLA-DR) and CD86, were specifically downregulated upon mAb P-3E10 ligation. The MHC class I (HLA-ABC), however, was not altered by mAb P-3E10. These results suggest that mAb P-3E10 could induce T cell unresponsiveness by interfering with the expression of cell surface molecules and subsequently disrupting the signal transduction of CD4^+^ T cells. Downregulation of co-stimulatory molecules on monocytes via the specific mAbs or ligands leading to the induction of T cell hypofunction has been reported [11, 37–39].

Some monocyte cytokines can modify their surface molecule expression [40–42]. We speculated that cytokines generated by monocytes themselves might be implicated in the negative regulation of the expression of MHC class II and CD86 molecules by mAb P-3E10. We found that mAb P-3E10 could promote significantly elevated numbers of monocytes producing IL-6, IL-10 and TNF-α. IL-6 treatment has been demonstrated to reduce HLA-DR and CD86 expression, but does not alter HLA-ABC and CD80 on human monocyte derived dendritic cells leading to attenuating IFN‑γ production by CD4^+^ T cells [43]. TNF-α was shown as an IL-10 inducer resulting in strongly increased IL-10 mRNA expression and IL-10 secretion in human monocytes [44]. IL-10 and TNF-α have been implicated in downregulation of MHC class II and CD86 on monocytes and macrophages resulting in the reduction of T cell proliferation and cytokine production [40, 45–48]. We, therefore, hypothesized that the binding of Na, K ATPase β3 subunit on monocytes by mAb P-3E10 promoted the increase of IL-6, IL-10 and TNF-α production. These cytokines then affect the down regulation of MHC class II and CD86 co-stimulatory molecules on monocytes leading to the suppression of T cell activation and Th1, Th2 and Th17 cytokine production.

Currently, human monocytes can be classified into classical (CD14^++^CD16^-^), intermediate (CD14^++^CD16^+^) and non-classical (CD14^+^CD16^++^) subsets [49, 50]. By flow cytometry analysis, the expression of Na, K ATPase β3 subunit was found in a homogeneous pattern among the monocyte population [13, 51]. The results suggested that all monocyte subsets might express the Na, K ATPase β3 subunit on their surface in the same manner. Upon stimulation, different monocyte subsets produce different cytokines. TNF-α, IL-6, IL-1β and IL-10 have been reported to be produced differently among monocyte subsets [45, 46, 50]. In response to TLR agonists, classical monocyte subsets are the most producers for TNFα, IL-6 and IL-1β and non-classical monocytes are the lowest producers [50]. Nevertheless, the classical monocytes produce high levels of IL-10 and low levels of TNF-α whereas intermediate monocytes mainly releasing TNF-α have been controversially reported [52, 53]. In this study, mAb P-3E10 induced IL-6, IL-10 and TNF-α production in monocytes. The monocyte subsets that produce cytokines in response to mAb P-3E10, however, are still unknown. Specifying which monocyte subsets are responsible for the cytokine production is of interest for further investigation. Recently, the mechanism of TNF-α induced IL-10 upregulation in monocytes was demonstrated. Autocrine binding of TNF-α secreted by LPS-stimulated classical monocytes upregulated IL-10 via tumor necrosis factor receptor 2 (TNFR2) [54]. The produced IL-10 and TNF-α decreased cytokine production of T cells has been documented [40, 45–48]. We therefore postulated that ligation of Na, K ATPase β3 subunit on monocytes by mAb P-3E10 enhances monocyte cytokine production which then affects T cell proliferation and cytokine production. The mAb P-3E10 clearly had no direct effect on T cells as no inhibitory effect of mAb P-3E10 in purified T cells were observed.

Various cytokines secreted by effector T cells have been reported to be increased and mediate several autoimmune and inflammatory diseases. Increased levels of Th1 and Th17 cytokines are associated with several inflammatory diseases including rheumatoid arthritis, multiple sclerosis and systemic lupus erythematosus [55–57]. The increase of Th2 cytokine is related to autoimmune thyroiditis and systemic lupus erythematosus progression [56, 58]. T cell activation and cytokine production is associated with an increased expression of MHC class II, CD86, CD80 and CD40 on monocytes/macrophages at the local inflammatory site in autoimmune patients [55, 57]. Blocking co-stimulatory pathways to induce T cell anergy is suggested as a potential treatment for autoimmune disease [9–12]. The blockade of co-stimulatory pathways for reducing T cell responses by chimeric proteins or antibodies has been employed as a short-term therapeutic approach. The benefit of short-term treatment is that T cells can be re-activated to maintain host defense against infection [9, 10]. In this present report, we demonstrated that mAb P-3E10 has the ability to mediate T cell hypofunction by manipulating monocyte activity. This mAb, however, does not have any effect on the function of Na, K ATPase activity [15]. The mAb P-3E10 might be a promising novel immunotherapeutic antibody for the short-term blockade of monocytes to impair T cell activation in autoimmune and inflammatory diseases.

## Supporting information

S1 Fig Validation of the specificity of purified mAb P-3E10. (A) Wild type and Na, K ATPase β3 subunit expressing BW5147 cells were stained with purified mAb P-3E10 (white peak with solid line) and mAb 13M (isotype-matched control mAb; gray peak with dotted line) by immunofluorescence technique. The specificity of mAb P-3E10 was analyzed by flow cytometry. (B) PBMCs were stained with purified mAb P-3E10 (black) and mAb 13M (white) by immunofluorescence technique. The specificity of mAb P-3E10 was analyzed by flow cytometry.
(PDF)

S2 Fig The inhibition of T cell cytokine production by mAb P-3E10. PBMCs were activated with anti-CD3 mAb OKT3 or kept unstimulated in the presence of mAb P-3E10 or mAb 13M (isotype-matched control mAb). The representative flow cytometric data from one of the three individuals were expressed in dot plot showing the percentage of the indicated cytokine producing T cells in the indicated conditions.
(PDF)

S3 Fig Ligation of monocytes by mAb P-3E10 regulates T cell activation. (A) PBMCs and monocyte-depleted PBMCs were activated with anti-CD3 mAb or kept unstimulated (medium alone) in the absence or presence of mAb P-3E10 or isotype-matched control mAb. (B) Purified T cells and purified T cells co-cultured with autologous purified monocytes were activated with anti-CD3 mAb (and anti-CD28 mAb) or kept unstimulated (medium alone) in the absence or presence of mAb P-3E10 or isotype-matched control mAb. (C) Monocytes were pre-pulsed with mAb P-3E10 or isotype-matched control mAb or medium before adding to purified T cells. Cells were activated with anti-CD3 mAb or kept unstimulated (medium alone). (D) THP1-cells were pre-pulsed with mAb P-3E10 or isotype-matched control mAb or medium. The pre-pulsed THP1 cells were co-cultured with PBMCs and activated with anti-CD3 mAb or kept unstimulated. Flow cytometric data were expressed in dot plot showing the percentage of the CD69 and CD25 expressing T cells in the indicated conditions. (E) Purified T cells were co-cultured with autologous purified monocytes either in the same well (together) or in separate compartments in a 96-transwell plate (separately). Cells were activated with anti-CD3 mAb or kept unstimulated (medium alone) in the absence or presence of mAb P-3E10 or isotype-matched control mAb. (A-C, E) Flow cytometric data were expressed in histograms showing the percentage of divided cells in each condition using CFSE proliferation assay.
(PDF)

S4 Fig Ligation of Na, K ATPase β3 subunit on monocytes by mAb P-3E10 downregulates MHC class II and CD86 expressions. (A) PBMCs were stimulated with anti-CD3 mAb in the absence (Medium) or presence of mAb P-3E10 (P-3E10) or isotype-matched control mAb (Isotype). The surface expression levels of MHC class I (HLA-ABC), MHC class II (HLA-DR) and CD86 on CD14+ monocytes were exhibited in over layered histograms in the presence of indicated conditions.
(PDF)
