# Supplementary material for: Ligation of Na, K ATPase β3 subunit on monocytes by a specific monoclonal antibody mediates T cell hypofunction
Source: PLoS One. 2018 Jun 25;13(6):e0199717. doi: 10.1371/journal.pone.0199717 (PMC6016913; doi:10.1371/journal.pone.0199717)
Supplement: S1 Fig — (A) Wild type and Na, K ATPase β3 subunit expressing BW5147 cells were stained with purified mAb P-3E10 (white peak with solid line) and mAb 13M (isotype-matched control mAb; gray peak with dotted line) by immunofluorescence technique. The specificity of mAb P-3E10 was analyzed by flow cytometry. (B) PBMCs were stained with purified mAb P-3E10 (black) and mAb 13M (white) by immunofluorescence technique. The specificity of mAb P-3E10 was analyzed by flow cytometry. (PDF) [file pone.0199717.s001.pdf]

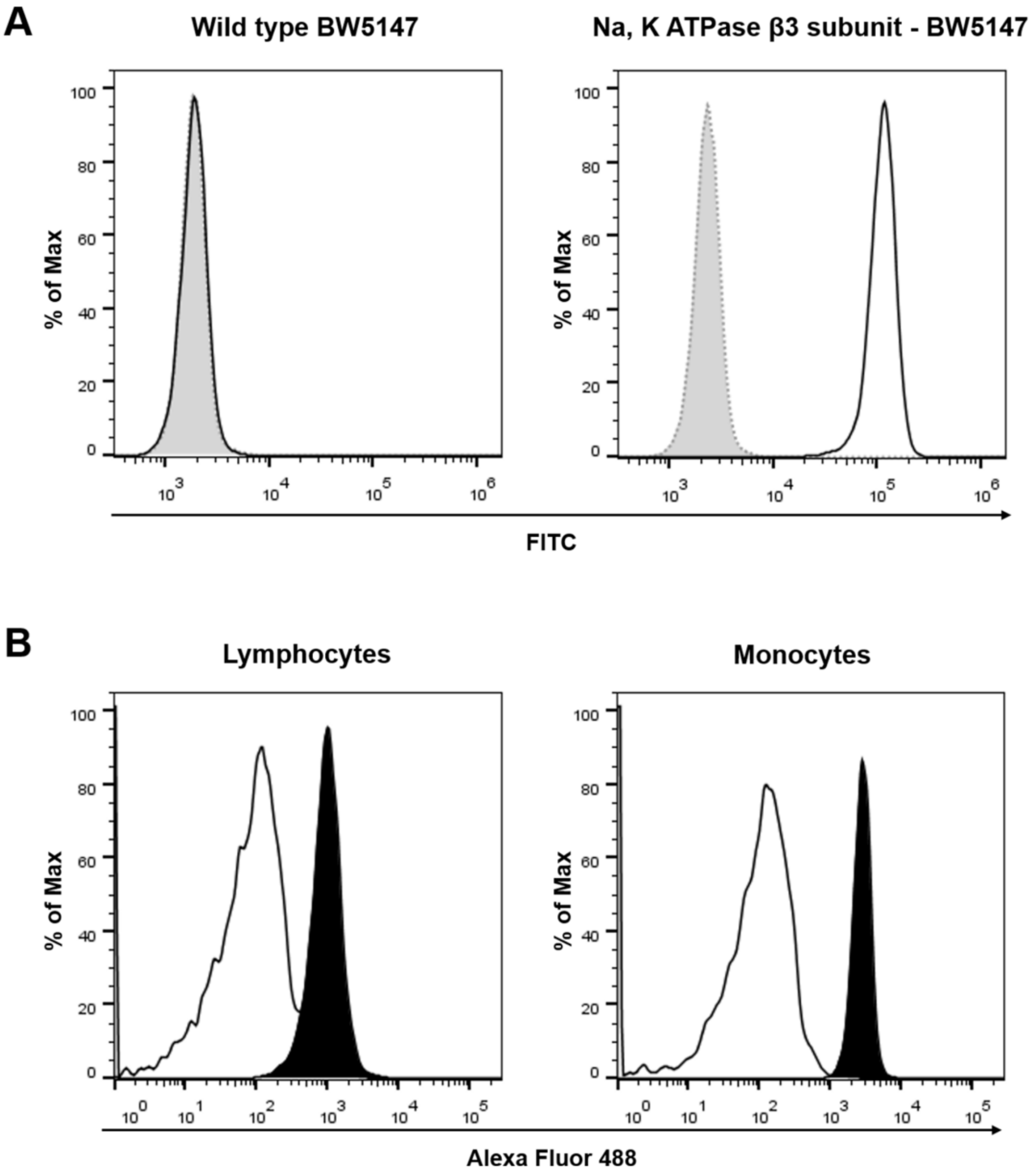

S1 Fig. Validation of the specificity of purified mAb P-3E10. (A) Wild type and Na, K ATPase β3 subunit expressing BW5147 cells were stained with purified mAb P-3E10 (white peak with solid line) and mAb 13M (isotype-matched control mAb; gray peak with dotted line) by immunofluorescence technique. The specificity of mAb P-3E10 was analyzed by flow cytometry. (B) PBMCs were stained with purified mAb P-3E10 (black) and mAb 13M (white) by immunofluorescence technique. The specificity of mAb P-3E10 was analyzed by flow cytometry.
