# Supplementary material for: Ligation of Na, K ATPase β3 subunit on monocytes by a specific monoclonal antibody mediates T cell hypofunction
Source: PLoS One. 2018 Jun 25;13(6):e0199717. doi: 10.1371/journal.pone.0199717 (PMC6016913; doi:10.1371/journal.pone.0199717)
Supplement: S2 Fig — PBMCs were activated with anti-CD3 mAb OKT3 or kept unstimulated in the presence of mAb P-3E10 or mAb 13M (isotype-matched control mAb). The representative flow cytometric data from one of the three individuals were expressed in dot plot showing the percentage of the indicated cytokine producing T cells in the indicated conditions. (PDF) [file pone.0199717.s002.pdf]

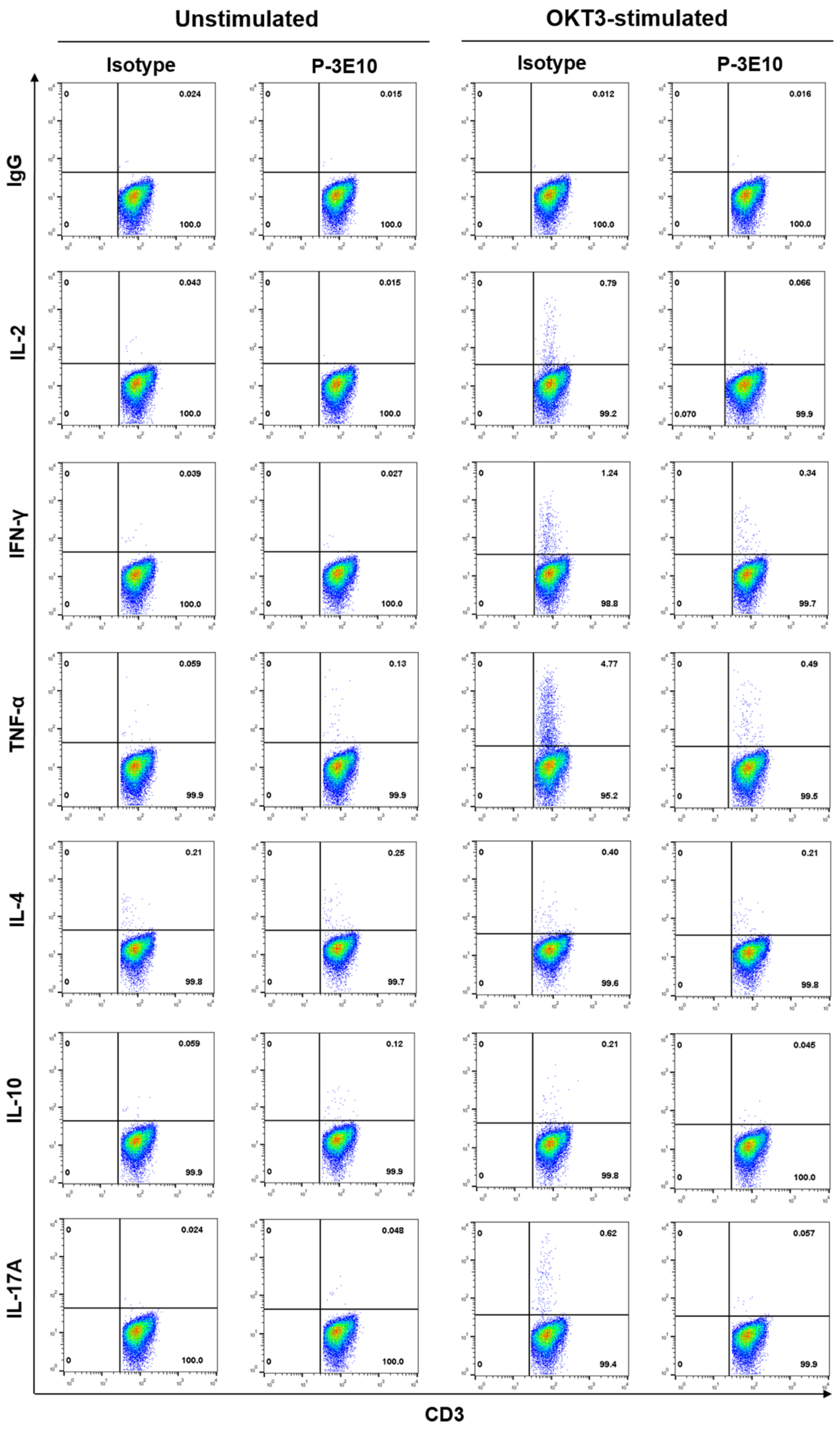

Unstimulated
OKT3-stimulated
Isotype
P-3E10
Isotype
P-3E10
IgG
IL-2
IFN-γ
TNF-α
IL-4
IL-10
IL-17A
CD3
0.024
100.0
0.015
100.0
0.012
100.0
0.016
100.0
0.043
100.0
0.015
100.0
0.79
99.2
0.066
0.070
99.9
0.039
100.0
0.027
100.0
1.24
98.8
0.34
99.7
0.059
99.9
0.13
99.9
4.77
95.2
0.49
99.5
0.21
99.8
0.25
99.7
0.40
99.6
0.21
99.8
0.059
99.9
0.12
99.9
0.21
99.8
0.045
100.0
0.024
100.0
0.048
100.0
0.62
99.4
0.057
99.9

S2 Fig. The inhibition of T cell cytokine production by mAb P-3E10. PBMCs were activated with anti-CD3 mAb OKT3 or kept unstimulated in the presence of mAb P-3E10 or mAb 13M (isotype-matched control mAb). The representative flow cytometric data from one of the three individuals were expressed in dot plot showing the percentage of the indicated cytokine producing T cells in the indicated conditions.
