# Supplementary material for: Ligation of Na, K ATPase β3 subunit on monocytes by a specific monoclonal antibody mediates T cell hypofunction
Source: PLoS One. 2018 Jun 25;13(6):e0199717. doi: 10.1371/journal.pone.0199717 (PMC6016913; doi:10.1371/journal.pone.0199717)
Supplement: S3 Fig — (A) PBMCs and monocyte-depleted PBMCs were activated with anti-CD3 mAb or kept unstimulated (medium alone) in the absence or presence of mAb P-3E10 or isotype-matched control mAb. (B) Purified T cells and purified T cells co-cultured with autologous purified monocytes were activated with anti-CD3 mAb (and anti-CD28 mAb) or kept unstimulated (medium alone) in the absence or presence of mAb P-3E10 or isotype-matched control mAb. (C) Monocytes were pre-pulsed with mAb P-3E10 or isotype-matched control mAb or medium before adding to purified T cells. Cells were activated with anti-CD3 mAb or kept unstimulated (medium alone). (D) THP1-cells were pre-pulsed with mAb P-3E10 or isotype-matched control mAb or medium. The pre-pulsed THP1 cells were co-cultured with PBMCs and activated with anti-CD3 mAb or kept unstimulated. Flow cytometric data were expressed in dot plot showing the percentage of the CD69 and CD25 expressing T cells in the indicated conditions. (E) Purified T cells were co-cultured with autologous purified monocytes either in the same well (together) or in separate compartments in a 96-transwell plate (separately). Cells were activated with anti-CD3 mAb or kept unstimulated (medium alone) in the absence or presence of mAb P-3E10 or isotype-matched control mAb. (A-C, E) Flow cytometric data were expressed in histograms showing the percentage of divided cells in each condition using CFSE proliferation assay. (PDF) [file pone.0199717.s003.pdf]

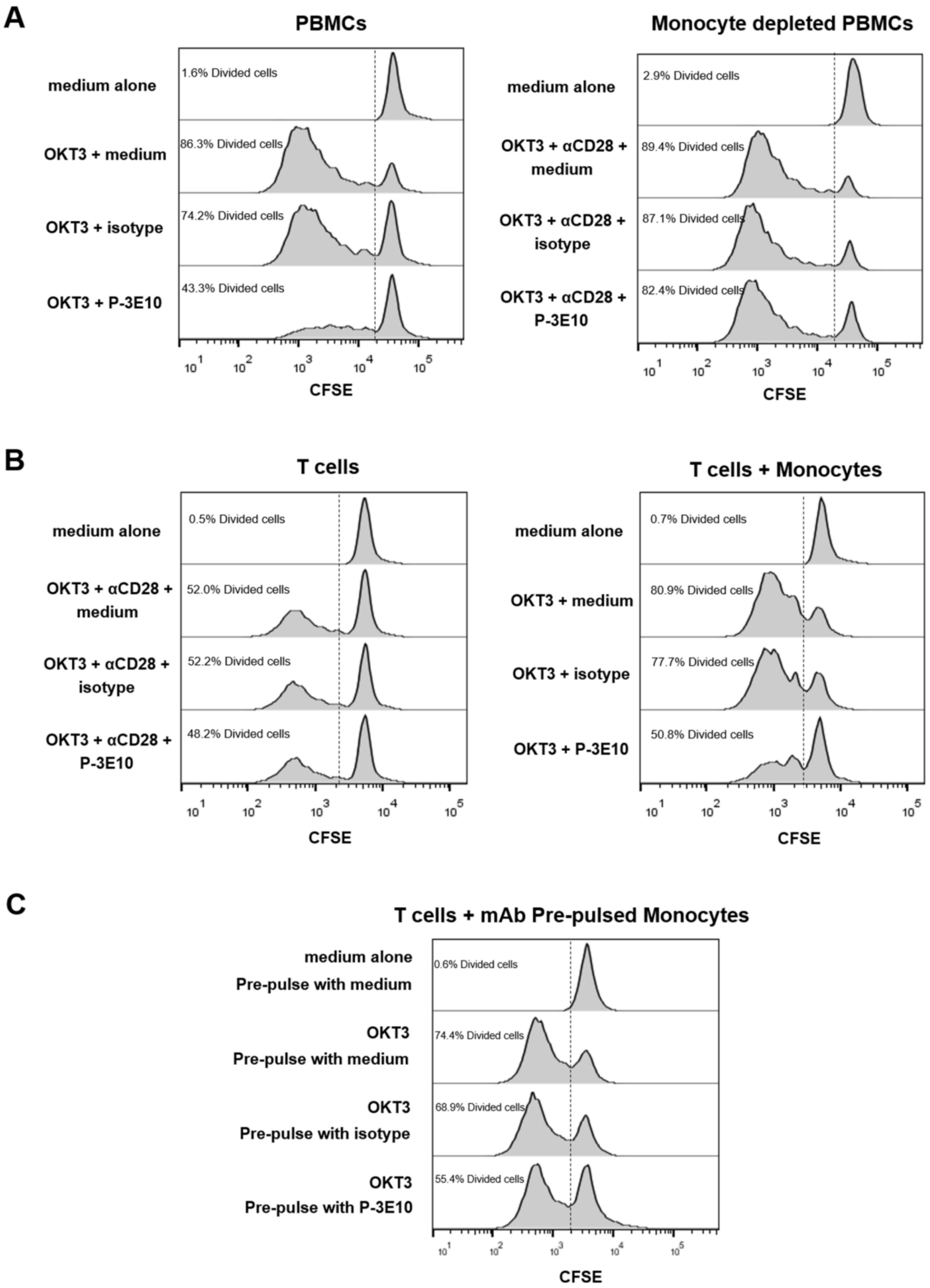
A
PBMCs
medium alone
1.6% Divided cells
OKT3 + medium
86.3% Divided cells
OKT3 + isotype
74.2% Divided cells
OKT3 + P-3E10
43.3% Divided cells
CFSE
Monocyte depleted PBMCs
medium alone
2.9% Divided cells
OKT3 + αCD28 + medium
89.4% Divided cells
OKT3 + αCD28 + isotype
87.1% Divided cells
OKT3 + αCD28 + P-3E10
82.4% Divided cells
CFSE
B
T cells
medium alone
0.5% Divided cells
OKT3 + αCD28 + medium
52.0% Divided cells
OKT3 + αCD28 + isotype
52.2% Divided cells
OKT3 + αCD28 + P-3E10
48.2% Divided cells
CFSE
T cells + Monocytes
medium alone
0.7% Divided cells
OKT3 + medium
80.9% Divided cells
OKT3 + isotype
77.7% Divided cells
OKT3 + P-3E10
50.8% Divided cells
CFSE
C
T cells + mAb Pre-pulsed Monocytes
medium alone
Pre-pulse with medium
0.6% Divided cells
OKT3
Pre-pulse with medium
74.4% Divided cells
OKT3
Pre-pulse with isotype
68.9% Divided cells
OKT3
Pre-pulse with P-3E10
55.4% Divided cells
CFSE

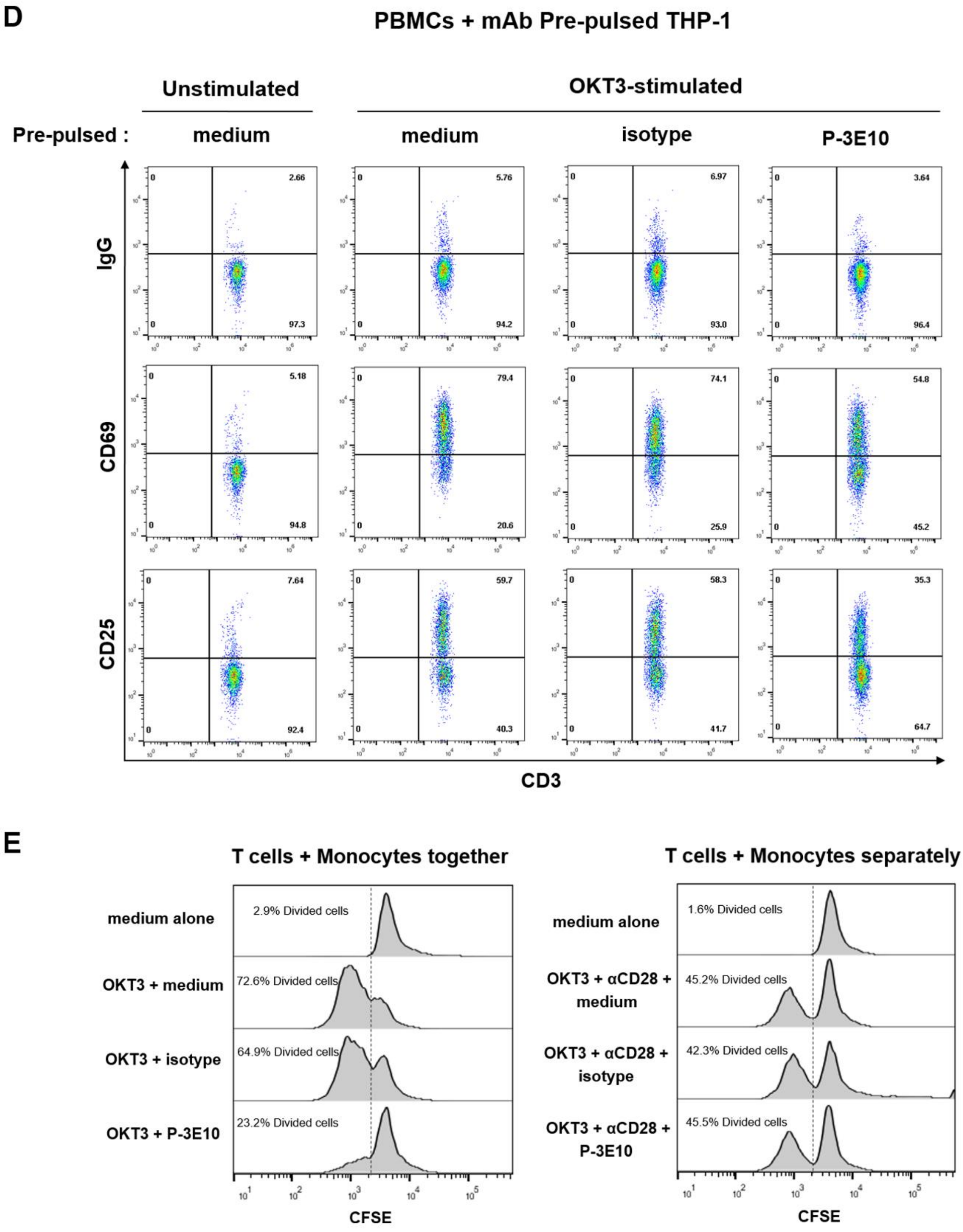

S3 Fig. Ligation of monocytes by mAb P-3E10 regulates T cell activation. (A) PBMCs and monocyte-depleted PBMCs were activated with anti-CD3 mAb or kept unstimulated (medium alone) in the absence or presence of mAb P-3E10 or isotype-matched control mAb. (B) Purified T cells and purified T cells co-cultured with autologous purified monocytes were activated with anti-CD3 mAb (and anti-CD28 mAb) or kept unstimulated (medium alone) in

the absence or presence of mAb P-3E10 or isotype-matched control mAb. (C) Monocytes were pre-pulsed with mAb P-3E10 or isotype-matched control mAb or medium before adding to purified T cells. Cells were activated with anti-CD3 mAb or kept unstimulated (medium alone). (D) THP1-cells were pre-pulsed with mAb P-3E10 or isotype-matched control mAb or medium. The pre-pulsed THP1 cells were co-cultured with PBMCs and activated with anti-CD3 mAb or kept unstimulated. Flow cytometric data were expressed in dot plot showing the percentage of the CD69 and CD25 expressing T cells in the indicated conditions. (E) Purified T cells were co-cultured with autologous purified monocytes either in the same well (together) or in separate compartments in a 96-transwell plate (separately). Cells were activated with anti-CD3 mAb or kept unstimulated (medium alone) in the absence or presence of mAb P-3E10 or isotype-matched control mAb. (A-C, E) Flow cytometric data were expressed in histograms showing the percentage of divided cells in each condition using CFSE proliferation assay.
