# Supplementary material for: Ligation of Na, K ATPase β3 subunit on monocytes by a specific monoclonal antibody mediates T cell hypofunction
Source: PLoS One. 2018 Jun 25;13(6):e0199717. doi: 10.1371/journal.pone.0199717 (PMC6016913; doi:10.1371/journal.pone.0199717)
Supplement: S4 Fig — (A) PBMCs were stimulated with anti-CD3 mAb in the absence (Medium) or presence of mAb P-3E10 (P-3E10) or isotype-matched control mAb (Isotype). The surface expression levels of MHC class I (HLA-ABC), MHC class II (HLA-DR) and CD86 on CD14+ monocytes were exhibited in over layered histograms in the presence of indicated conditions. (PDF) [file pone.0199717.s004.pdf]

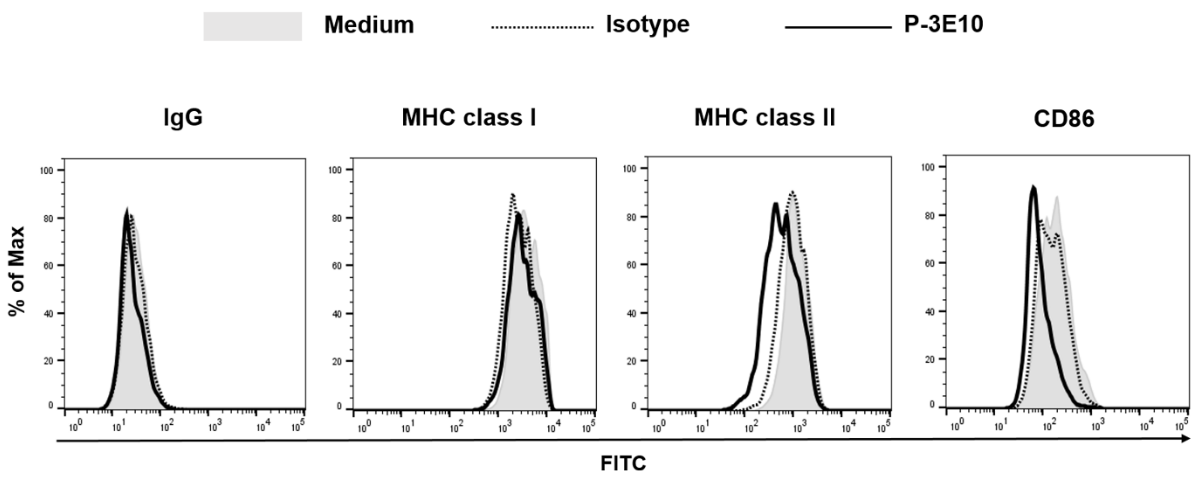

S4 Fig. Engagement of Na, K ATPase β3 subunit on monocytes by mAb P-3E10 downregulates MHC class II and CD86 expressions. (A) PBMCs were stimulated with anti-CD3 mAb in the absence (Medium) or presence of mAb P-3E10 (P-3E10) or isotype-matched control mAb (Isotype). The surface expression levels of MHC class I (HLA-ABC), MHC class II (HLA-DR) and CD86 on CD14+ monocytes were exhibited in over layered histograms in the presence of indicated conditions.
